# Examining Recruits in Buffalo

**Published:** 1863-05

**Authors:** 


### EDITORIAL DEPARTMENT.

### EXAMINING RECRUITS IN BUFFALO.

Great complaints have been made in times past that recruits for the volunteer service were examined and passed by incompetent surgeons, and hence that the service suffered by having its hospitals filled by men who should never have been received into it. Men with infirmities which were disqualifying and which should have been discovered, were passed only to break down at the first trial of endurance. In these cases however, even at that early period, such incompetency or negligence was for the most part brought to the doors of *medical* men. We rejoice to believe that in these early days of want of proper care, it was to no great extent the habit to go out of the profession and take up irregulars and quacks to sit in judgment upon the physical qualifications necessary to make a man a good soldier and fit for enlistment. Even officers anxious to secure their commissions by getting the requisite number of recruits, without being scrupulous as to their physical soundness, for the most part, sought *medical* men, if not of the first quality. At this time, to the honor of the service, and of those most anxious to swell its numbers merely, a due sense of the importance of examinations by competent surgeons, has become almost universal, especially among those who have been in service and have seen the evils of careless or ignorant inspection.

The latter, particularly, have been actuated by a high and honorable sense of duty to the service, deeming any want of precaution in the nature of a breach of trust. What shall we say then when we find the mustering officer in this City, swearing men into the service upon the certificate as to their physical soundness, of a man not only, not a regular physician, but, if we are correctly informed, not a physician at all, i. e. not a Doctor of Medicine, and besides a practitioner of homœopathy? We can call it by no other name than laxity in the discharge of a duty to which he was assigned by the military authorities with, no doubt, full confidence that he would be guided by a strict regard to the interests of the government and the efficiency of our army. It certainly seems very much like preferring his own convenience to the Nation's good. If it is not a forgetfulness of obligation and indifference to duty, the most charitable view we can take of the matter is, that it is an inability to appreciate the importance of the trust committed to him. It is mortifying to our pride that, here in Buffalo, where there are so many men in the profession competent for such thorough examination of enlisted men, as the regulations of the army require, it should be left to one, who is not recognized as one of its members. We certainly never expected at this late day of the war, and after the experience of needless expense and crippled strength which has been so painfully manifest heretofore, arising from laxity, to call it by no harder name, similar to that of which we now complain; we repeat, we certainly never expected to find the necessity which we now find, for calling attention to an abuse so deserving of censure and so damaging to the efficiency of the military service. We regret also to hear that an agent of the State of Massachusetts—a State which has in this war been distinguished for sending into the service medical men of great excellence—employs the same man to act as examining surgeon for recruits forwarded from this City to the 54th (black) regiment now in camp at Readville, Mass. We are not interested to save the State of Massachusetts the expense of returning to Buffalo men who, though passed here, will be found unfit for service when they arrive at camp, but we are interested that whatever pertains to the medical or surgical service of our army, should be entrusted to competent, regular physicians and surgeons. Probably the course taken was due to the fact that the aforesaid homœopathist was found charged with the examinaination of recruits by the mustering officer here, and that fact alone, without special enquiry being taken, was sufficient guaranty that he was a competent and proper person for the service required. The surgeon of the regiment would, however, have saved himself from a liability of being charged with not exercising due diligence in the matter, had he put himself in communication with some one or more members of the regular profession, who would have willingly given him such information as would have enabled him to decide whom not to employ, as well as whom to employ.

